# Simultaneous Determination of 8 Compounds in Gancao-Ganjiang-Tang by HPLC-DAD and Analysis of the Relations between Compatibility, Dosage, and Contents of Medicines

**DOI:** 10.1155/2017/4703632

**Published:** 2017-05-14

**Authors:** Yanfang Yang, Guijun Zhang, Qiyu Sun, Liang Liu, Hui Peng, Jingjuan Wang, Li Xiang

**Affiliations:** ^1^Department of Pharmacognosy, Beijing University of Chinese Medicine, Beijing 100102, China; ^2^Zibo Wanjie Institute of Traditional Chinese Medicine, Shandong 255000, China

## Abstract

Gancao-Ganjiang-Tang (GGT) is a traditional Chinese medicine (TCM) prescription and is a representative prescription for recuperating depleted Yang in Treatise on Febrile Diseases. The TCM theory believes that the efficacy of medicinal herbs is decided by the multicompounds which consist of different kinds of chemical constituents with bioactivities, but not by a monomeric constituent. From ancient times until today, GGT have 5 different kinds of compatibilities that can be verified. In this study, a HPLC-DAD method was established for the simultaneous determination of 8 compounds including 6-gingerol, 8-gingerol, 6-shogaol, liquiritin, liquiritigenin, isoliquiritin, isoliquiritigenin, and glycyrrhizic acid in the five GGT. The total contents of the 8 compounds in GGT varied from 555.56 to 956.33 *μ*g/mL. The effects showed that the dosage and compatibility of medicinal herbs have influenced the content of chemical compounds of TCM prescription while the content of chemical compounds has acted on clinical efficacy. Quality evaluation and active essence screening of TCM (including single herb and prescription) should be based on the TCM theory and clinical effectiveness. The method was proven to be suitable for quality control of GGT.

## 1. Introduction

Gancao-Ganjiang-Tang (GGT), a traditional Chinese medicine (TCM) prescription from the* Treatise on Febrile Diseases*, was a combination of the crude extracts from Glycyrrhizae Radix and Rhizoma Zingiberis and was used as a “warming” preparation for patients with chills, fatigue, dysphoric, and so forth caused by Yang deficiency [1]. GGT contains lots of chemical constituents and produces curative effects through synergic reaction. 6-Gingerol, 8-gingerol, 6-shogaol, liquiritin, liquiritigenin, isoliquiritin, isoliquiritigenin, and glycyrrhizic acid are the most important bioactive constituents reported at present. Glycyrrhizic acid can exert anti-inflammation effect by suppressing cyclooxygenase-2 (COX-2) and inducible nitric oxide synthase (iNOS) expression [2]; it also has other effects including immunomodulatory [3], antidiabetic effect [4], liver protection [5], inhibits cholestasis [6], and neuroprotection [7]. Liquiritigenin has the effects in reducing the expression of promatrix metalloproteinase-2 [8] and inhibiting hypoxia-inducible factor 1*α* (HIF-1*α*) and vascular endothelial growth factor (VEGF) expression [9]. Isoliquiritigenin belongs to chalcone compound and has valuable biological activities such as anticancer, antioxidant, anti-inflammatory, and antiallergic activities. liquiritin and isoliquiritin have the pharmacological properties of antimicrobial [10], antioxidative [11], antitumor [12], and antiulcer activities [13] and others. Gingerols are a mixture and are the spicy ingredients in Rhizoma Zingiberis as well as the main active ingredients, among them are 6-gingerol, 8-gingerol, and 6-shogaol which are most outstanding. Gingerols can enhance power of cardiac muscle systole and have a protective effect on lipid peroxidation damage of nervous cell membrane induced by radicals; gingerols can also effectively protect the activity of catalase in the ischemia reperfusion brain tissue of rats while simultaneously ameliorating the ischemia and anoxic state in tissue, and so forth [14]. All the compounds mixed together make the decoction have good efficacy in complex diseases.

Form ancient times, the combination of Rhizoma Zingiberis and Glycyrrhizae Radix has the remarkable curative effect to cold syndrome, such as epigastralgia, vomit acid saliva, diarrhea, back and chest pain, vertigo, and asthmatic cough, caused by Yang-qi deficiency and yin excessiveness [15]. The therapeutic rules for the Yang depletion are warming of the weakened organs and recuperating depleted Yang to save the body from collapsing [1]. The structure of Glycyrrhizae Radix and Rhizoma Zingiberis is a fundamental prescription for recuperating depleted Yang in TCM theories.

According to clinical symptoms, GGT have significant differences in compatibility and dosage (Table 1). For example, in Japan, GGT is composed of Glycyrrhizae Radix, Rhizoma Zingiberis, Fructus Zanthoxyli, and Aconiti Lateralis Radix Praeparata. It has been described in Shan-qi-zheng-zhi-lun by Da-qiao-shang-yin in the Meiji Restoration, which is widely applied for the treatment of hernia symptoms and diarrhea. Similarly, GGT consists of Glycyrrhizae Radix, Rhizoma Zingiberis, and Schisandrae Chinensis Fructus in Xue-lun-zheng written by Rongchuan Tang and is aimed specifically at treating failure of spleen to control circulating blood. According to the theories of monarch drugs, minister drugs, assistant drugs, and guide drugs in TCM theories [16], the compatibility and dose of GGT have changed, but the Rhizoma Zingiberis and Glycyrrhizae Radix still dominate the curative powers.

TCM (including single herb and prescription) is a complex system, which contains many compounds. When single herb is used in combination, these compounds in each single herb will influence each other and then treat complex diseases together. Moreover, in TCM prescription, the change of dosage and compatibility of single herb may produce different symptoms. Now the quality control of TCM focuses on single herb or the monomeric compound, which cannot show the treatment effects accurately, cannot reflect the interactions between constituents, and cannot ensure the safety and efficacy of TCM. For example, both Chrysanthemi Flos and Lonicerae Japonicae Flos contain chlorogenic acid. Chlorogenic acid is regarded as marker for quality control of them in Chinese pharmacopoeia [17]. However, they are two different kinds of TCM with different clinical application.

Evaluating the quality of TCM should be based on TCM theory. The TCM theory believes the efficacy of TCM is decided by the multicompounds which consist of many different kinds of chemical constituents with bioactivities, but not by a monomeric constituent. In this study, a HPLC-DAD method was developed to analyze multicompounds of GGT including 6-gingerol, 8-gingerol, 6-shogaol, liquiritin, liquiritigenin, isoliquiritin, isoliquiritigenin, and glycyrrhizic acid for exploring a comprehensive quality control criterion for TCM.

## 2. Materials and Methods

### 2.1. Chemicals and Reagents

The reference standards of 6-gingerol, 8-gingerol, 6-shogaol, liquiritin, liquiritigenin, isoliquiritin, isoliquiritigenin, and glycyrrhizic acid (purity ≥ 98%) were purchased from Chengdu Pufeide Biotech Co., Ltd. (Chengdu, China). Methanol, acetonitrile (Fisher, USA), and phosphoric acid (Tianjin Chemical Regent Co., Ltd., Tianjin, China) were of HPLC grade. The distilled water was obtained from Wahaha Co., Ltd. (Hangzhou, China).

Medicinal materials were purchased from Hebei Anguo medicine market (Hebei, China) and authenticated by Professor Guijun Zhang (Beijing University of Chinese Medicine, Beijing, China).

### 2.2. Instrumentation and Separation Conditions

A thermo 3000 liquid chromatography system (Thermo, USA), equipped with a quaternary solvent delivery system, a DAD detector, an autosampler, a column heater, and a Hypersil Gold-C_18_ (4.6 mm × 250 mm, 5 *μ*m) column, was used. The mobile phase consisted of (A) acetonitrile and (B) 0.1% H_3_PO_4_ aqueous (V/V). Optimum separation was obtained by using a gradient elution described in Table 2. The flow rate was 0.6 mL·min^−1^ and injection volume was 20 *μ*L. The column temperature was set at 30°C and the wavelengths were shown in Table 3.

### 2.3. Sample Preparation

TCM prescriptions (Table 1) were immersed with 10-fold volume of water and boiled twice for 30 min at each time, followed by filtration. The filtrates from each prescription were merged and concentrated under reduced pressure to 1/2 of its original volume. For HPLC analysis, 2 mL of filtrate was dissolved in methanol at a volume of 10 mL and then centrifuged with a rate of 100000/min for 10 min. The supernatant was filtered through a filter (0.22 *μ*m pore size) prior to injection. And the negative control groups were prepared in the same manner.

### 2.4. Preparation of Standard Solution

The appropriate amount of 6-gingerol, 8-gingerol, 6-shogaol, liquiritin, liquiritigenin, isoliquiritin, isoliquiritigenin, and glycyrrhizic acid was weighed and dissolved in methanol to achieve eight standard working solutions separately; the concentrations of the eight reference compounds were 1700 *μ*g/mL, 870 *μ*g/mL, 960 *μ*g/mL, 740 *μ*g/mL, 810 *μ*g/mL, 850 *μ*g/mL, 770 *μ*g/mL, and 1110 *μ*g/mL. The calibration curves were constructed by analyzing the eight standard solutions and the series of working solutions within the ranges of 10.625~170 *μ*g/mL for 6-gingerol, 5.4375~87 *μ*g/mL for 8-gingerol, 0.75~96 *μ*g/mL for 6-shogaol, 18.5~296 *μ*g/mL for liquiritin, 5.0625~81 *μ*g/mL for liquiritigenin, 7.9688~127.5 *μ*g/mL for isoliquiritin, 0.9625~77 *μ*g/mL for isoliquiritigenin, and 69.375~1110 *μ*g/mL for glycyrrhizic acid, respectively.

### 2.5. Method Validation

The HPLC method was employed and methodology was examined for linearity, recovery, precision, repeatability, and stability. The validation was implemented based upon the relative peak areas. Linear regression analysis was used to prepare calibration curves. And relative standard deviation (RSD) was used to evaluate precision, repeatability, stability, and recovery.

### 2.6. Statistical Analysis

Results were expressed as means ± standard deviation (SD). Differences in mean values between groups were analyzed by a one-way analysis of variance (ANOVA). Statistical significance was considered at *P* < 0.05.

## 3. Results

### 3.1. Chromatographic Conditions

**Figure 1:**
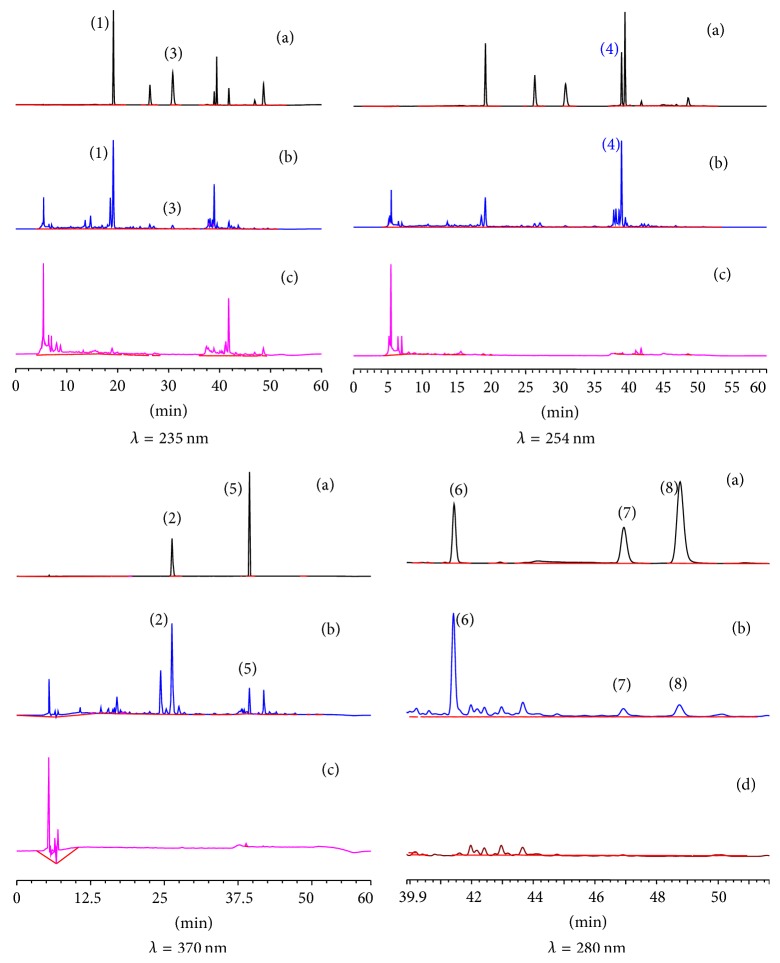
HPLC chromatograms of standard mixture (a), GGT test sample (b), test sample without Radix Glycyrrhizae (stir-baked with honey) (c), and test sample without Rhizoma Zingiberis (d). (1) Liquiritin, (2) isoliquiritin, (3) liquiritigenin, (4) glycyrrhizic acid, (5) isoliquiritigenin, (6) 6-gingerol, (7) 8-gingerol, and (8) 6-shogaol.

Acetonitrile (A) and 0.1% H_3_PO_4_ aqueous (B) (V/V) were chosen as the composition of mobile phases for all the analyses. The chromatogram of mixed standard compounds, GGT sample, and negative control sample were shown in Figure 1. In Figure 1, the eight peaks marked with 1–8 are liquiritin, isoliquiritin, liquiritigenin, glycyrrhizic acid, isoliquiritigenin, 6-gingerol, 8-gingerol, and 6-shogaol. The retention time is 19.133 min, 26.300 min, 30.767 min, 38.937 min, 39.487 min, 41.820 min, 46.797 min, and 48.557 min, respectively. The negative control sample had no peaks at the corresponding positions of GGT sample, which illustrated the other medicine herbs did not interfere the determination.

### 3.2. Calibration Curves

Using the above chromatographic conditions, the calibration curves of 8 compounds exhibited good linear regressions. The calibration curves were constructed by plotting the peak area and the corresponding concentration of the compounds in the freshly prepared plasma calibrators.  Table 4 gives the linear regression equation, linear range, limit of quantitation (LOQs), and limit of detections (LODs) of eight standard substances.

### 3.3. Precision, Repeatability, Stability, and Recovery

The precision was obtained by six copies of determinations individually of the standard solution. The repeatability was performed by six-time determinations continuously of a sample. Stability was tested with GGT sample solution and standard solution that were stored at room temperature at several time points (0, 2, 4, 8, 12, and 24 h after preparation), and the 8 compounds were found to be rather stable within 24 h (RSD < 3%; *n* = 6). In the recovery test, samples were prepared at three concentration levels in triplicate by spiking known quantities of each of the 8 standards into the GGT sample and then extracted and analyzed according to the described procedures. All of these data are shown in Table 5.

### 3.4. Contents of Eight Compounds in GGT

**Figure 2:**
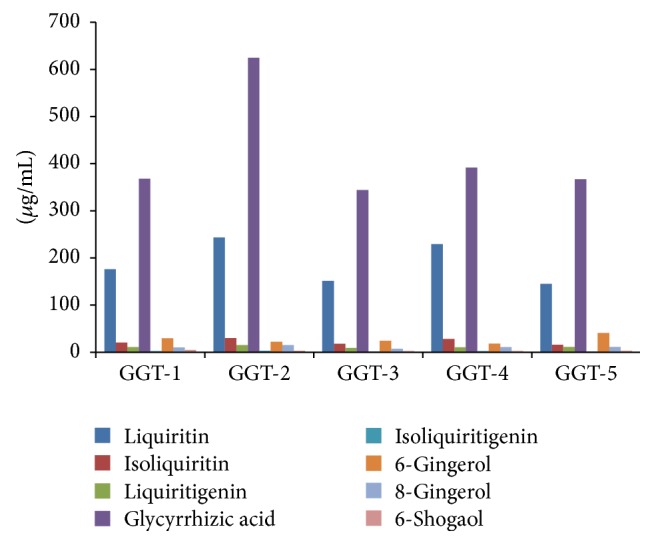
The contents of eight compounds in GGT (*μ*g/mL) (*n* = 3).

The HPLC data illustrated that different compatibility of medicinal herbs has different contents. The material foundation of medicinal herbs is a combination of numerous active components following some complicated theorems (linear and nonlinear), rather than an element or a kind of element, or the simple sum of these elements. The content of eight compounds in GGT is shown in Table 6 and Figure 2.

## 4. Discussion

### 4.1. HPLC Conditions

Compared with acetonitrile and 0.05% phosphoric acid aqueous (V/V) and acetonitrile and 0.1% acetic acid aqueous (V/V), the separation ability of acetonitrile and 0.1% phosphoric acid aqueous (V/V) system is higher. The result showed that separation of peak 5 and peak 6 is not very good in 35°C and separation of peak 2 is not very good in 25°C. By comparison, 30°C will be just right. The flow rate of 0.6 mL/min was determined by testing different flow rates. When the flow rate is too high, such as 0.8 mL/min and 1.0 mL/min, peaks cannot be separated from peaks. When the flow rate is too slow, the whole retention times were extended while the resolutions remained the same. The final gradient elution method was decided by testing various gradient elution methods.

### 4.2. Selections of Eight Active Compounds

The TCM theory believes that the efficacy of TCM is due to the multicompounds which consist of many different kinds of chemical constituents. Moreover, research and development of the multicompounds in TCM tend to be a direction of TCM modernization because of their therapeutic and pharmaceutical advantages. 6-Gingerol, 8-gingerol, and 6-shogaol are the major active components of Rhizoma Zingiberis [14]. Liquiritin, liquiritigenin, isoliquiritin, isoliquiritigenin, and glycyrrhizic acid are the major active components of Radix Glycyrrhizae [18]. The eight components of GGT have clear chemical structure, obvious pharmaceutical properties, and convenient detection, so they should be considered for study first.

### 4.3. Correlation between Compatibility, Dosage, and Content of Medicinal Herbs

According to the holistic concept and syndrome differentiation theories, it is a common phenomenon to change the TCM prescription (including compatibility and dosage) to accommodate symptoms. Meanwhile the compatibility and dosage of TCM prescription have an impact on its content of chemical compositions.

When the dosage of TCM prescription keeps constant and the compatibility of prescription changed, the content of components is changing with compatibility consequently. Take GGT-1 and GGT-2, for example: GGT-1 is composed of Rhizoma Zingiberis (6 g, raw) and Radix Glycyrrhizae (12 g, stir-baked with honey), and GGT-2 is composed of Rhizoma Zingiberis (6 g, stir-baked) and Radix Glycyrrhizae (12 g, stir-baked with honey). The two prescriptions have same dosage but different medicinal herbs. We can see from Table 6 that the total content of eight compounds of GGT-1 and GGT-2 had obvious difference and the difference was statistically significant (*P* < 0.05), which indicated that the compatibility has an impact on the content of medicinal herbs. Therefore GGT-1 and GGT-2 have different contents of chemical compounds. The contents are (*μ*g/mL) 175.68 ± 0.932, 20.23 ± 0.305, 10.87 ± 0.068, 367.79 ± 0.987, 1.99 ± 0.018, 29.48 ± 0.114, 9.93 ± 0.034 and 4.88 ± 0.029, and 243.14 ± 0.699, 29.87 ± 0.108, 14.97 ± 0.163, 624.53 ± 0.997, 3.29 ± 0.012, 22.42 ± 0.242, 14.97 ± 0.162, and 3.14 ± 0.022, respectively.

When the compatibility of TCM prescription keeps constant and the dosage of TCM prescription changed, the content of components is changing with dosage consequently. Take GGT-2 and GGT-3, for example: GGT-2 is composed of Rhizoma Zingiberis (6 g, stir-baked) and Radix Glycyrrhizae (12 g, stir-baked with honey), and GGT-3 is composed of Rhizoma Zingiberis (9 g, stir-baked) and Radix Glycyrrhizae (9 g, stir-baked with honey). The two prescriptions have the same compatibility but different dosage level. Similarly, the total content of eight compounds of GGT-2 and GGT-3 had obvious difference and the difference was statistically significant (*P* < 0.05), which indicated that the dosage has an impact on the content of medicinal herbs. Therefore GGT-2 and GGT-3 have the different contents of chemical compounds. The contents are (*μ*g/mL) 243.14 ± 0.699, 29.87 ± 0.108, 14.97 ± 0.163, 624.53 ± 0.997, 3.29 ± 0.012, 22.42 ± 0.242, 14.97 ± 0.162 and 3.14 ± 0.022, and 151.42 ± 0.865, 17.45 ± 0.092, 8.92 ± 0.053, 344.03 ± 1.212, 1.52 ± 0.031, 24.209 ± 0.346, 7.17 ± 0.049, and 0.84 ± 0.015, respectively.

From the above analysis, we can know when the compatibility and dosage of TCM prescription changed simultaneously and the content of components are changing consequently. Like with GGT-4 and GGT-5, GGT-4 is composed of Rhizoma Zingiberis (6 g, stir-baked), Radix Glycyrrhizae (15 g, stir-baked with honey), and Schisandrae Chinensis Fructus (3 g, raw), and GGT-5 is composed of Rhizoma Zingiberis (15 g, raw), Radix Glycyrrhizae (15 g, raw), Fructus Zanthoxyli (9 g, raw), and Aconiti Lateralis Radix Praeparata (9 g). The two prescriptions have different compatibility and dosage level, and the effective substances of them are different: The contents of GGT-4 are (*μ*g/mL) 229.05 ± 0.829, 28.06 ± 0.42, 10.28 ± 0.114, 391.56 ± 1.632, 2.23 ± 0.011, 17.99 ± 0.068, 10.89 ± 0.052 and 0.97 ± 0.014; GGT-5 are (*μ*g/mL) 144.81 ± 0.687, 15.96 ± 0.212, 11.23 ± 0.086, 366.93 ± 1.23, 1.86 ± 0.045, 40.89 ± 0.92, 11.07 ± 0.058, and 1.29 ± 0.011.

### 4.4. Correlation between Compatibility, Dosage of Medicinal Herbs, and Symptoms

**Figure 3:**
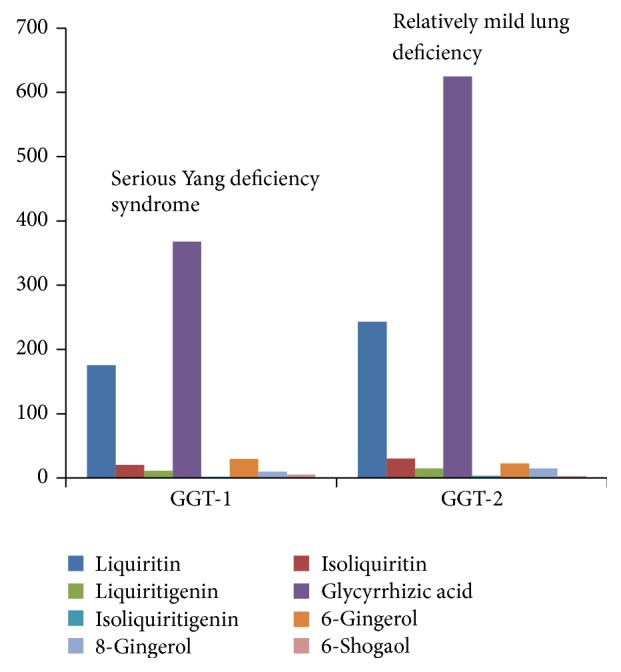
Correlation between symptoms and content of GGT-1 and GGT-2.

**Figure 4:**
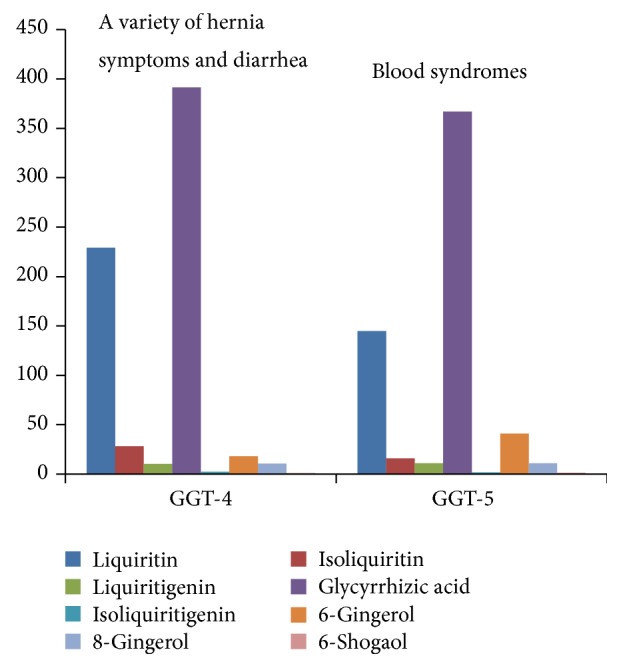
Correlation between symptoms and content of GGT-4 and GGT-5.

**Figure 5:**
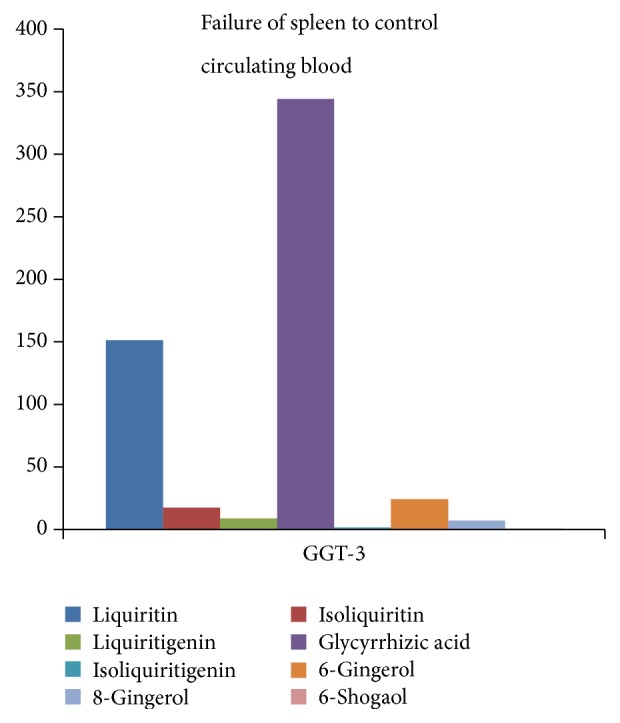
Correlation between symptoms and content of GGT-3.

Traditional Chinese medicine decoction contains many compositions and various physicochemical actions coexist and interact. These compositions formed a relatively stable combination with a certain components and these components have a fixed ratio in content. This is why TCM prescription of different compatibility and dosage are being used to treat different diseases. The conclusion has been already proved and recorded in the medical classical documents written by medical experts at home and abroad in ancient times. GGT-1 is used to treat serious Yang deficiency syndrome, and symptoms include spontaneous perspiration, chills, dry throat, leg cramps, dizziness, vomit, and frequent urination, in Shang-han-lun by Zhongjing Zhang, while GGT-2 is applied to relatively mild lung deficiency, and symptoms include cough, dyspnea, and asthenia, in Jin-kui-yao-lue. And the composition ratios of them are 88 : 10 : 5 : 184 : 1 : 15 : 5 : 2.5 and 77 : 9.5 : 5 : 199 : 1 : 7 : 5 : 1, separately (Figure 3). GGT-4 is applied to failure of spleen to control circulating blood caused by spleen-Yang deficiency in Xue-lun-zheng by Rongchuan Tang; and GGT-5 has specific effects and great power for a variety of hernia symptoms and diarrhea in Shan-qi-zheng-zhi-lun by Japanese scholar. And the composition ratios of them are 236 : 29 : 11 : 404 : 2 : 19 : 11 : 1 and 112 : 12 : 9 : 284 : 1 : 32 : 9 : 1, separately (Figure 4). GGT-3 is often used for blood syndromes, such as epistaxis, woman bleeding, and hematemesis in Ren-zhai-zhi-zhi-fang-lun by Shilei Yang. The composition ratio of GGT-3 is 180 : 21 : 11 : 410 : 2 : 29 : 9 : 1 (Figure 5).

## 5. Conclusions

TCM is the important part of Chinese culture, through thousands of years' development, having formed a self-contained system and becoming the typical representative of eastern medicine. We should develop an effective and reasonable method to monitor the quality of TCM from a whole perspective. This study provides a new HPLC method for quality control of the GGT. The result showed that GGT mentioned above are different in compatibility and dosage, and the indications of them are different too. The method could be suitable for quality control of GGT with bioactive multicompounds.

## Figures and Tables

**Table 1:** Compatibility of medicines, dosage, and symptoms of GGT in different period.

Reference	Period	Compatibility of TCM	Dosage	Symptoms
Shang-han-lun (GGT-1)	Han dynasty	Rhizoma Zingiberis (raw) and Radix Glycyrrhizae (stir-baked with honey)	12 g, 6 g	Serious Yang deficiency syndrome

Jin-kui-yao-lue (GGT-2)	Han dynasty	Rhizoma Zingiberis (stir-baked) and Radix Glycyrrhizae (stir-baked with honey)	12 g, 6 g	Relatively mild lung deficiency

Ren-zhai-zhi-zhi-fang-lun (GGT-3)	Song dynasty	Rhizoma Zingiberis (stir-baked) and Radix Glycyrrhizae (stir-baked with honey)	9 g, 9 g	Failure of spleen to control circulating blood caused by spleen-Yang deficiency

Xue-lun-zheng (GGT-4)	Qing dynasty	Rhizoma Zingiberis (stir-baked), Radix Glycyrrhizae (stir-baked with honey), and Schisandrae Chinensis Fructus (raw)	15 g, 6 g, 3 g	A variety of hernia symptoms and diarrhea

Shan-qi-zheng-zhi-lun (GGT-5)	Meiji restoration period (Japan)	Rhizoma Zingiberis (raw), Radix Glycyrrhizae (raw), Fructus Zanthoxyli (raw), and Aconiti Lateralis Radix Praeparata	15 g, 15 g, 9 g, 9 g	Blood syndromes

**Table 2:** The binary gradient elution system.

Time (min)	0~5	5~10	10~15	15~30	30~32	32~35	35~40	40~45
A: acetonitrile (%)	5~15	15~22	22~26	26~28	28~55	55~60	60~63	63~65

**Table 3:** Detection wavelengths.

Wavelengths (nm)	235 nm	254 nm	280 nm	370 nm
Compounds	Liquiritin Liquiritigenin	Glycyrrhizic acid	6-Gingerol 8-Gingerol 6-Shogaol	Isoliquiritin Isoliquiritigenin

**Table 4:** Linear regression equation, linear range, LOD, and LOQ for eight standard substances.

Standard substance	Regression equation	Correlation coefficient (*r*^2^)	Linear range (*μ*g/mL)	LOD (*μ*g/mL)	LOQ (*μ*g/mL)
Liquiritin	*y* = 0.2557*x* − 0.5111	0.9998	18.5~296	1.52	4.56
Isoliquiritin	*y* = 1.6975*x* − 2.2464	0.9999	7.9688~127.5	0.512	1.536
Liquiritigenin	*y* = 1.3507*x* − 1.1905	0.9997	5.0625~81	0.477	1.431
Glycyrrhizic acid	*y* = 0.2929*x* − 3.2464	0.9998	69.375~1110	2.561	7.683
Isoliquiritigenin	*y* = 2.8843*x* − 0.2564	0.9997	0.9625~77	0.193	0.574
6-gingerol	*y* = 0.2292*x* − 0.69	0.9995	10.625~170	1.847	5.541
8-Gingerol	*y* = 0.2742*x* − 0.6137	0.9997	5.4375~87	1.013	3.039
6-Shogaol	*y* = 2.4176*x* − 0.6731	0.9996	0.75~96	0.132	0.396

**Table 5:** Precision, repeatability, stability, and recovery of eight substances.

Compound	Precision RSD (%) (*n* = 6)	Repeatability RSD (%) (*n* = 6)	Stability RSD (%) (*n* = 6)	Recoveries (%)^*∗*^	
				(*n* = 9)	RSD (%)
Liquiritin	0.88	1.36	0.51	99.89	1.58
Isoliquiritin	1.02	1.19	0.79	101.67	1.28
Liquiritigenin	1.73	0.97	1.32	98.93	1.33
Glycyrrhizic acid	0.99	1.82	0.33	100.87	0.97
Isoliquiritigenin	1.67	1.01	0.95	101.26	2.87
6-Gingerol	1.58	1.96	2.11	99.94	1.45
8-Gingerol	1.29	1.49	1.46	100.74	1.76
6-Shogaol	1.42	2.01	0.72	98.75	2.83

^*∗*^Recovery (%) = 100 *∗* (amount found − original amount)/amount spiked.

**Table 6:** The contents of eight compounds in GGT (*n* = 6).

Compound	*C* (*μ*g/mL)				
	GGT-1	GGT-2	GGT-3	GGT-4	GGT-5
Liquiritin	175.68 ± 0.932	243.14 ± 0.699	151.42 ± 0.865	229.05 ± 0.829	144.81 ± 0.687
Isoliquiritin	20.23 ± 0.305	29.87 ± 0.108	17.45 ± 0.092	28.06 ± 0.42	15.96 ± 0.212
Liquiritigenin	10.87 ± 0.068	14.97 ± 0.163	8.92 ± 0.053	10.28 ± 0.114	11.23 ± 0.086
Glycyrrhizic acid	367.79 ± 0.987	624.53 ± 0.997	344.03 ± 1.212	391.56 ± 1.632	366.93 ± 1.230
Isoliquiritigenin	1.99 ± 0.018	3.29 ± 0.012	1.52 ± 0.031	2.23 ± 0.011	1.86 ± 0.045
6-Gingerol	29.48 ± 0.114	22.42 ± 0.242	24.21 ± 0.346	17.99 ± 0.068	40.89 ± 0.920
8-Gingerol	9.93 ± 0.034	14.97 ± 0.162	7.17 ± 0.049	10.89 ± 0.052	11.07 ± 0.058
6-Shogaol	4.88 ± 0.029	3.14 ± 0.022	0.84 ± 0.015	0.97 ± 0.014	1.29 ± 0.011
Total content of eight compounds	620.85 ± 1.42^*∗*^	956.33 ± 2.744	555.56 ± 1.23^*∗*^	691.03 ± 3.232	594.04 ± 3.41

^*∗*^Compared with GGT-2, *P* < 0.05.
